# Residual bone growth after lengthening procedures

**DOI:** 10.1007/s11832-016-0792-y

**Published:** 2016-11-23

**Authors:** Pierre Journeau, Pierre Lascombes, Dominique Barbier, Dmitry Popkov

**Affiliations:** 1 Paediatric Orthopaedic Surgery Department, Nancy University Hospital Centre, Children’s Hospital, Vandoeuvre lès Nancy, France; 2 Paediatric Orthopaedic Surgery Department, Geneva University Hospitals, Children’s Hospital, Geneva, Switzerland; 3 Scientific and Clinical Laboratory for Deformity Correction and Limb Lengthening, Federal Russian State-Financed Ilizarov, Kurgan, Russia

**Keywords:** Lower limb length discrepancy, Progressive bone lengthening, Residual growth

## Abstract

The prognosis of limb length discrepancy is a major subject in paediatric orthopaedic surgery. The strategy depends on the prognosis and must be adapted to each patient. The residual growth of the lengthened segment often remains unknown, but is dependent on age, the percentage of lengthening and other factors. Using a large cohort of 150 children who had undergone bone lengthening procedures, we describe five patterns of post-intervention growth and identify factors that are favourable for normal residual growth. The criteria for bone lengthening which should maintain good residual growth are—bone age at lengthening should be before the pubertal growth spurt; the interval between two lengthening procedures should be over three years; the percentage of lengthening should be <30% of the initial segment; and no more than two lengthening procedures should be carried out during infancy.

## Introduction

Accurancy in predicting future lower limb length discrepancies in congenital abnormalities is a major challenge for paediatric orthopaedic surgeons and the primary question asked by parents. Several charts, such as Moseley, Hechard and Carlioz, allow a prediction of the possible final difference in length [1]. According to the predicted discrepancy, the strategy for correction is then adapted to the clinical situation and the parents’ wishes, and could call for progressive bone lengthening, transitory or definitive epiphysiodesis, orthosis or other procedures. However, little is known about the evolution of residual bone growth after progressive bone lengthening, and different patterns of slowing, accelerating or normal growth have all been reported independently of the lengthening techniques (Judet, Wagner, Callotasis, and Cauchoix) [2–10].

To try to understand the factors influencing residual growth after progressive lengthening, we studied a large cohort of children who had undergone progressive limb lengthening procedures [11].

## Series analysis

One hundred and fifty paediatric patients with congenital limb length discrepancy were studied and followed after progressive lengthening procedures until skeletal maturity. In total, these patients underwent 207 surgical segment lengthening procedures. In 42 cases, the segment was lengthened twice, and in 15 it was lengthened, three times. All the lengthened segments were monitored using Hechard and Carlioz charts, before and after the lengthening procedure, at 1–3 months, 6–8 months, 9–12 months and until skeletal maturity. Preoperatively, predicted limb length discrepancies and final segment lengths were assessed using the multiplier method [12], i.e., without treatment, the natural growth of an abnormal segment is linear, at a constant rate. In other words, the percentage of the discrepancy remains identical throughout the growth period [13].

In the series examined, the average gain in femoral length was 4.2 ± 1.43 cm or 17.3 ± 12.18% of the initial segment length. The average gain in tibial length was 4.8 ± 2.38 cm or 19.3 ± 11.04% of the initial segment length.

**Fig. 1:**
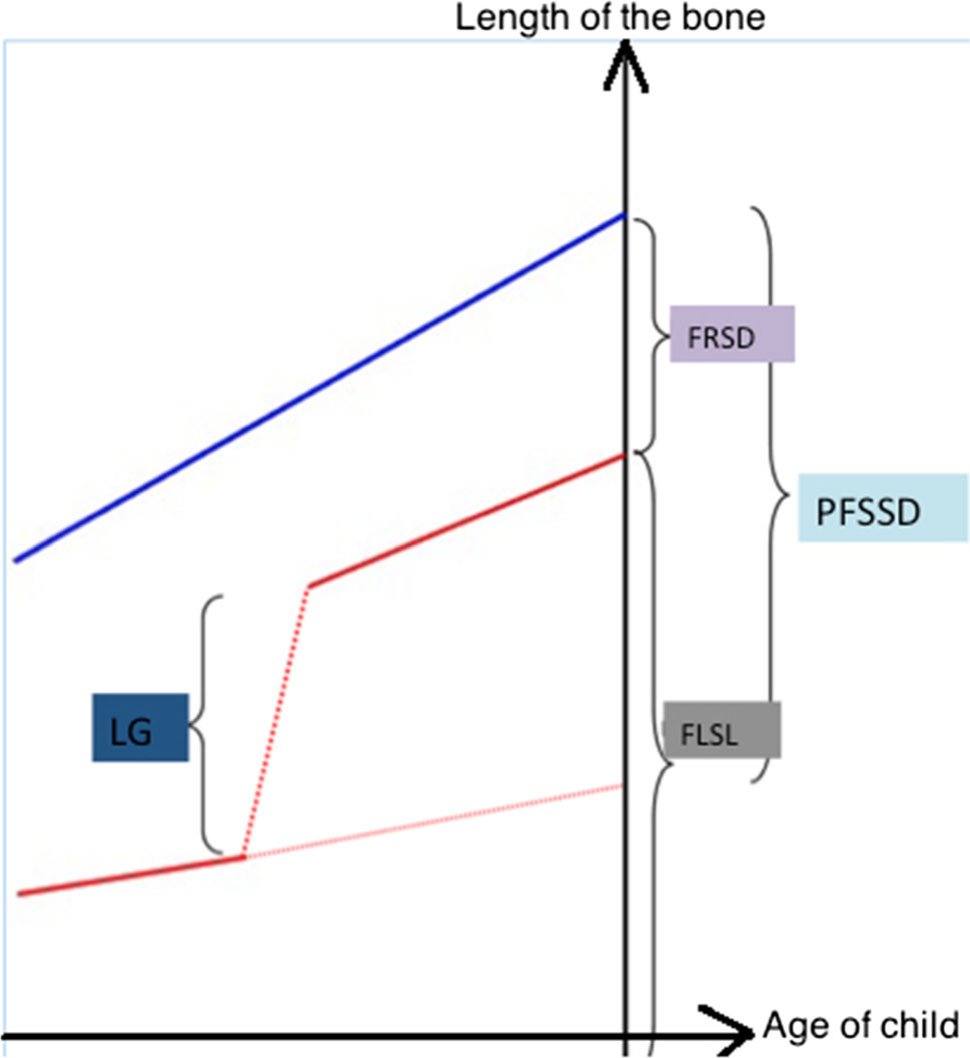
Diagram of the method for calculating residual growth gain. *PFSSD* predicted final spontaneous segmental discrepancy (mm), *LG* length gain achieved using external fixation (mm), *FRSD* final residual segmental discrepancy (mm; healthy segment length minus lengthened segment length), *FLSL* final lengthened segment length (mm)

During the follow-up, we investigated the different possible growth rate changes after lengthening, whether it appeared to have been stimulated, whether there was a transitory slowing of growth during the first year or whether there was a definitive arrest in growth. A specific new growth index was thus calculated at the end of the growth phase (Fig. 1):

$${\text{RGG }}\left( \% \right) = ({\text{PFSSD}} - ({\text{LG}} \pm {\text{FRSD}})) \times 100\% /{\text{FLSL}}$$

where, RGG = residual growth gain; PFSSD = predicted final spontaneous segmental discrepancy (mm); LG = length gain achieved using external fixation (mm); FRSD = final residual segmental discrepancy (mm; healthy segment length minus lengthened segment length); FLSL = final lengthened segment length (mm).

This index represents the change in natural growth after progressive lengthening with respect to the final length of the lengthened segment; it may be positive (accelerated growth), neutral (no change) or negative (slowdown in spontaneous growth).

**Fig. 2:**
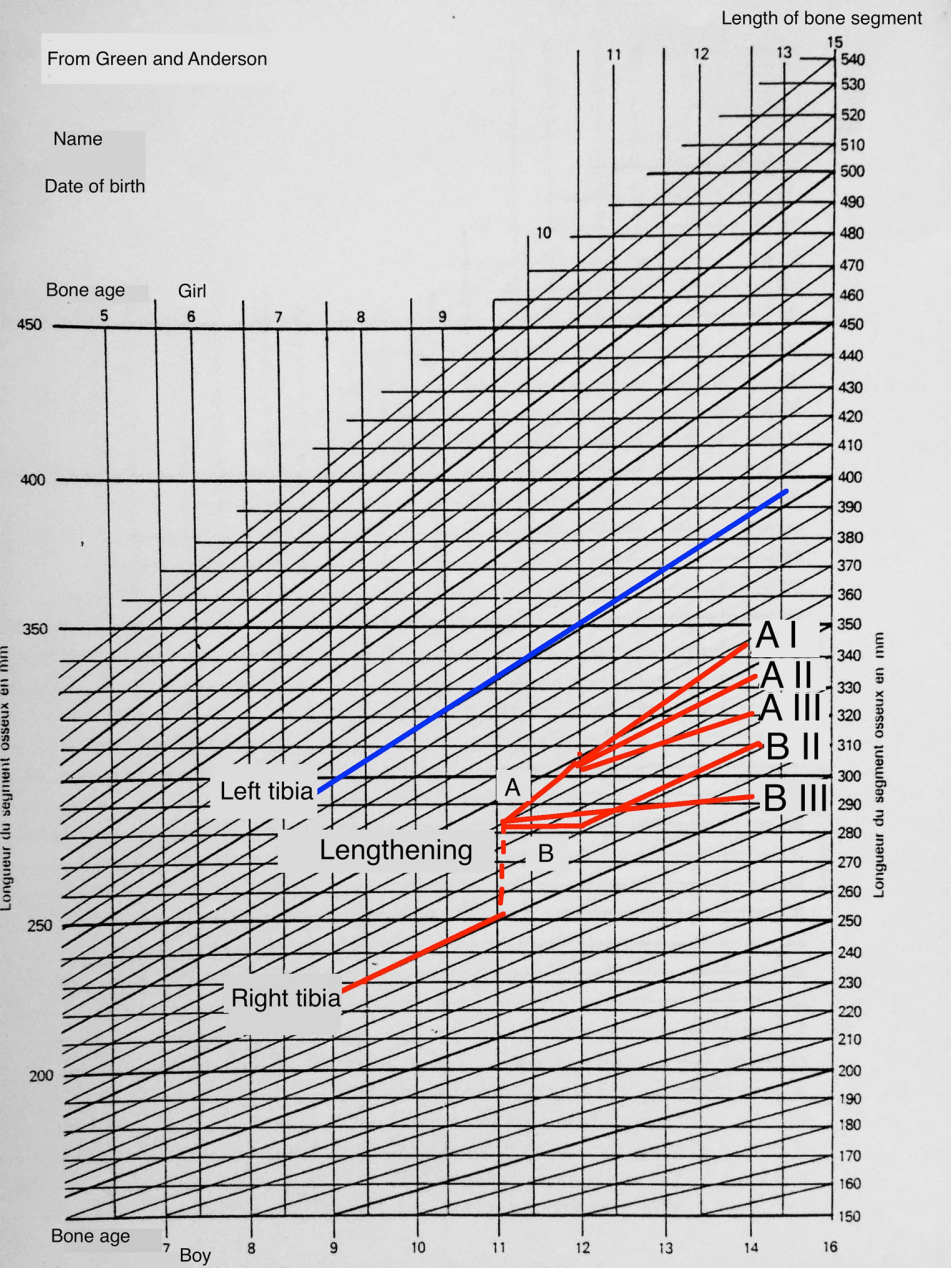
Diagram of five types of residual growth after lengthening

When observing longitudinal growth during follow-up, we distinguished and dichotomised changes in the operated segments into short-term (9–12 months follow-up) and long-term changes (>12 months) (Fig. 2):

- In the short-term, two groups were observed:
  - Group A = no change or a temporary increase in growth rate
  - Group B = transitory slowdown in growth rate
- In the long-term, three groups were observed:
  - Type I = acceleration of growth
  - Type II = growth identical to the preoperative rate
  - Type III = a slowdown in growth rate

This enabled us to describe six types of growth:

Group AI: no change or a transitory short-term increase in growth rate, followed by acceleration.

Group AII: no change or a transitory short-term increase in growth rate, followed by a return to the preoperative growth rate.

Group AIII: no change or a transitory short-term increase in growth rate, followed by progressively slowing growth.

Group BI: transitory short-term slowdown in growth rate, followed by an acceleration in growth.

Group BII: transitory short-term slowdown in growth rate, followed by a return to the preoperative growth rate.

Group BIII: transitory short-term slowdown in growth, followed by a progressive slowdown until a definitive arrest to growth in the long-term.

No cases of BI-type growth were observed in our cohort.

This means that for groups AIII, BII and BIII the prognosis of the final discrepancy after lengthening will be higher than the initially predicted prognosis due to the slowing rate of growth following the lengthening.

Comparing the types of residual growth, no statistical differences were found in relation to sex, the type of segment lengthened (femoral or tibial) or the lengthening method. However, we distinguished four main factors that did influence residual growth:

- Factor 1: the number of lengthening procedures. All the patients who underwent two or more lengthenings of the same segment were in group B, whether it was femoral or tibial lengthening (Tables 1, 2); thus, triple lengthening of a segment during the period of growth is considered inadvisable.

**Table 1:** Distribution of femoral growth types according to the stage of lengthening; number of cases

Growth type	Primary lengthening	Second lengthening	Third lengthening
A I	49	1	0
A II	18	0	0
A III	12	3	0
B I	5	0	0
B II	6	20	7

**Table 2:** Distribution of tibial growth types according to the stage of lengthening; number of cases

Growth type	Primary lengthening	Second lengthening	Third lengthening
A I	59	0	0
A II	21	2	2
A III	5	0	0
B I	11	4	0
B II	8	24	11
- Factor 2: the delay between two procedures. When procedures are <3 years apart, the risk of a slowdown in residual growth is high; the recommended gap between procedures is at least 3 years.
- Factor 3: the bone age at first lengthening. Below a bone age of 8.5–9 years in girls and 12 years in boys, no inhibition of the longitudinal growth of the lengthened segment was observed. If needed, a second lengthening procedure could be performed before the pubertal growth spurt in order to prevent any inhibition of residual growth in the lengthened segment.
- Factor 4: the association with other bone procedures. If reconstructive foot surgery is performed during or after tibial lengthening, then the residual tibial growth rate is slower than preoperatively; this correlation was noticed immediately after the first lengthening.

In summary, factors favourable for preventing residual bone growth problems are a bone-age cut-off for starting a first lengthening procedure of <9 years of age for girls and <12 years of age for boys, with a restoration of the mechanical axis of lower limb at the same time. If a second procedure is needed, it should take place before the pubertal growth spurt or after that growth, with a gap of >3 years between the first and the second lengthening.

## Discussion

Due to its impact on family, function and school, the correction of lower limb length discrepancies could be performed early. However, as this study has shown, the influence of lengthening procedures on residual growth must be considered in decisions on the surgical strategy to adopt when faced with congenital abnormalities. The percentage of length discrepancy remains stable in congenital aetiologies, and we consider that changes in the growth rate following lengthening are due to modifications to the growth pattern of the bone.

We have shown that growth stimulation was systematic in the few weeks or months after the first lengthening, when bone age was <9 years in girls and <12 years in boys. The mechanism for this could be the increased vascularisation of the bone during lengthening [6]. Restoration of the mechanical axis at the same time as lengthening is also a good prognostic factor for growth stimulation [14–16]. It allows better weight-bearing and increases the quality of the regenerated bone and the number of cells in the physis [17, 18]. Some experimental studies have confirmed that dynamic loading with a normal axis of the lower limb increases the number of cells in the physis [19]. The absence of weight-bearing during lengthening decreases the growth rate. This could explain why growth is inhibited when tibial lengthening is associated with foot surgery.

Penneçot et al. [3] reported that progressive bone lengthening using a Judet distractor had a significant impact on the growth plate. The harmful effects of hyper-pressure on cartilage tissue, impaired vascularisation and shaft ischaemia may account for the subsequent slowdown in growth. Oostenbroek et al. recommended bridging the knee in order to obtain a subsequent stimulation in growth [19, 20], but we did not observe any statistically significant modification in residual growth in the knee joint distractions performed. On the other hand, knee distraction avoids joint contracture and dislocation.

Another factor unfavourable for residual growth was a percentage of lengthening >30% of the initial segmental length, as this led to a slowdown in residual growth or arrested growth [7, 11]. However, in an experimental study, Gang demonstrated that a 30% lengthening had no effect on the residual growth of the tibia [21].

The influence of age when first starting lengthening procedures has been noted by many authors [4, 6] who reported growth disorders around the age of puberty, but growth stimulation before the age of 6–8 years. The hypothetical slowdown in growth rate when lengthening is performed during puberty could be due to the fact that soft tissues fail to adapt quickly enough during the lengthening procedure. Indeed, during pubertal growth, the increase in bone growth leads to soft-tissue stress. If bone lengthening is performed at that time, the soft tissues cannot stretch enough and cannot keep up with the lengthening. For this reason, we think that a preoperative period of physiotherapy is necessary, for stretching the soft tissues and maintaining an adequate range of motion in the joints. This could be a favourable factor for good results.

The alternative would be to perform the second lengthening around the end of childhood growth; this would avoid more than two lengthening procedures during infancy and too short an interval between two procedures.

## Conclusion

The five residual growth patterns described in this study were dependent on certain factors that caused acceleration or, on the contrary, a slowdown in the rate of growth—age at the lengthening procedure, the percentage of lengthening and the minimum period between two lengthening procedures. Respecting these criteria produced optimal conditions for excellent residual growth after progressive segmental lower limb lengthening. Respecting these criteria also helped to avoid risks of a slowdown or a complete arrest in growth. If this is the case, however, the paediatric orthopaedic surgeon can propose a planned, multi-step lengthening programme.
